# Observations on Erysipelas Infantile

**Published:** 1817-12

**Authors:** R. Moulson

**Affiliations:** Physician to the Halifax General Dispensary


### *Observations on Erysipelas Infantile.* By R. MOULSON, M. D. Physician to the Halifax General Dispensary.

THE Cases of Erysipelas Infantile which fell under my immediate care when a pupil in London, having terminated fatally, and under every mode of treatment; I was not a little surprised to find, upon perusing Dr. Davis's Report of the Universal Dispensary, at page 276 in the last number of your Journal, that Erysipelas had proved so tractable a disease in his hands; viz. "Small doses of sulphate of magnesia with antimonial wine, and saline diaphoretics, usually carried off the disease, after the previous exhibition of one or two active purgatives with submuriate of mercury and scammony. A slight opiate at night was also serviceable."

Indeed, I can candidly assert, that the disease above-mentioned has caused me no small degree of uneasiness, particularly when, after consulting several eminent practitioners in London, and searching for the observations of those who had written upon the subject, I could not overcome the ravages of this infanticide. I conceive, that the cases of Erysipelas which came under Dr. Davis's notice must have been of a much milder form, than those which came under mine.* The disease to which I allude

* I should not omit to mention, that all the cases which came under my observation were within the month; all of which I examined after death is well described by Dr. Underwood;* and previous to my having examined any cases by dissection, bark and aromatics were liberally given, and spirituous fomentations were applied to the part affected; these failing, purgatives were administered with salines; and these not succeeding, a very opposite treatment was adopted, the consequence of examining the bodies of those dying of the disease.

As I have Dr. Underwood's Treatise in my possession, I thought it unnecessary to note down the appearance upon dissection, he having stated what I likewise found: his words are, "Upon examining several bodies after death, the contents of the belly have frequently been found glued together, and their surface covered with inflammatory exudation, exactly similar to that found in women who have died of puerperal fever, &c." p. 36. It was evident, then, that the disease, ab origine, was truly inflammatory; and dissection clearly evinced the active ravages inflammation had made, which could only have been counteracted by a very opposite practice.

Mr. Burns, in his Principles of Midwifery, page 495, says, that he has derived more advantage from calomel in such doses as to act upon the bowels, than from any other medicine. The treatment, as recommended by Dr. Armstrong in his valuable Practical Illustrations of Typhus, &c. pages 179 and 180, I have strictly followed, with the exception of inducing faintness by leeching, which I was afraid to do in such very young patients; and again the disease has baffled my endeavours. I was informed, that following up the disease with blisters was the treatment of a French practitioner; and where I applied several blisters upon the same child, it appeared, that those parts only which had been previously leeched, seemed to be benefitted, which I could not help attributing to the previous abstraction of blood; for I have seen a single leech remove the diffused redness of a part in a few hours.

Upon examining a child after death, which had been treated by frequently leeching the erysipelatous parts, and repeated doses of the submuriate of mercury, none of those sequelæ of inflammation appeared within or upon the intestines, all appeared quite natural. Viewing the appearances of bodies after death, after these different modes of practice, it appears rational to give the preference to the depleting in conjunction with the alterative

* In his Treatise on the Diseases of Children; 6th edit. p. 34 to 38.

plan as recommended by Dr. Armstrong, and that to be prosecuted vigorously for the first twenty-four hours from the attack; for unless a change for the better is within that time, I fear all future endeavours will avail but little.

I was informed by an intelligent person, that in two of the Lying-in Hospitals in London, this disease proves universally fatal; whether or no it be contagious, I cannot speak decisively, but am inclined to think it is. The warm bath I always found aggravated the sufferings of the unfortunate infants. Should any of your Correspondents be able to lay down a successful mode of practice in Erysipelas Infantile, I am sure the medical profession will feel greatly indebted to them.

*Halifax, Yorkshire, October* 13, 1817.

